# Gut microbial metabolites as a convergence point between autoimmunity and solid tumors

**DOI:** 10.1080/29933935.2025.2470805

**Published:** 2025-03-12

**Authors:** Anu Shibi Anilkumar, Sheena Mariam Thomas, Ramakrishnan Veerabathiran

**Affiliations:** Human Cytogenetics and Genomics Laboratory, Faculty of Allied Health Sciences, Chettinad Hospital and Research Institute, Chettinad Academy of Research and Education, Kelambakkam, India

**Keywords:** Gut microbiome, microbial metabolites, cancer, autoimmune disease

## Abstract

The human gut microbiome, a diverse community of trillions of microorganisms, is essential for controlling numerous bodily functions, such as metabolism, immune response, and epithelial barrier integrity. The gut microbiota comprises bacteria, viruses, fungi, and other microorganisms that affect human health, metabolic pathways, and immune responses. Dysbiosis, or the imbalance of gut microbial composition, has been linked to the pathogenesis of several ailments, including cardiovascular conditions, gastrointestinal conditions, allergies, obesity, autoimmune disorders, and tumors. The interaction between gut microbes and immune responses, mainly through Tregs cells and Th17 cells, underscores the microbiome’s function in immune regulation. Furthermore, gut microbial metabolites act as signaling molecules and substrates for metabolic processes, impacting autoimmune disorders and cancer development. Recent research highlights the microbiome’s potential role in cancer immunoediting, where gut microbial metabolites may either promote or suppress cancer progression by modulating inflammation and immunosuppression. This review delves into the critical functions of the gut microbiome, its influence on autoimmune disorders, and the emerging connection between gut microbial metabolites and cancer immunoediting, offering new insights into their impact on human health and disease.

## Introduction

The human body harbors trillions of diverse and dynamic microbial communities, collectively termed the “microbiome,” that are pivotal in controlling various physiological processes.^63^ The gut microbiome, comprising bacteria, viruses, fungi, protozoa, and archaea, is especially notable, with around 100 trillion microorganisms in the gut alone. The gut microbiome is known to encode over three million genes and produce a vast array of metabolites, many of which complement or replace essential functions of the human host.^89^ The connection between gut microbiota and the host significantly impacts health, with metabolic byproducts having beneficial or detrimental effects. By colonizing intestinal surfaces, these microbes form a robust barrier, preventing harmful pathogens from entering the system.^79^ Additionally, the gut microbiome’s function, makeup, and variability are shaped by dietary habits, with varying diets playing a significant role in determining the stability and functionality of this microbial ecosystem.^76^

While microbial communities colonize various parts of the human body, the gut hosts the largest and most studied microbial population.^78^ Although bacterial colonization begins in the lower uterus, the gut microbiota primarily develops after birth, highlighting its crucial role in health.^59^ The gut microbiome has been implicated in modulating nutritional effects, both directly and indirectly, which underscores its vital role in host metabolism and overall health.^91^ The microbiome also influences critical biological processes, including immune system regulation, metabolic modulation, and the enhancement of epithelial barrier function.^63^ Genetics, gender, age, socioeconomic status, nutrition, stress, and environmental factors like pollutants and antibiotics are among the many factors that shape the gut microbiota composition. This composition plays a significant role in developing the host defense system and the effectiveness of immune reactions.^91^ The gut microbial community is crucial for the appropriate advancement of the defense system and plays a substantial role in the neurological and endocrine systems and in maintaining a healthy metabolism.^12^

Moreover, the microbiota residing in the gut is critical in regulating the host system’s metabolism and modulating immune responses and brain function.^25^ When the balance of gut microbiota is disrupted, a condition known as dysbiosis occurs, leading to various diseases such as cardiovascular disorders,^13^ gastrointestinal issues,^14^ allergies,^5^ obesity,^60^ and central nervous system-related conditions.^34^ Additionally, the intestinal flora significantly impacts the formation of gastrointestinal mucosal immunity, a fundamental part of our body’s immune defense system.^61^ Autoimmune disorders and cancer are two prominent disease families that stem from different expressions of immunological dysfunction.^62^ Autoimmune disorders, in particular, are marked by a dysregulated immune system that fails to distinguish between Host antigens and non-host antigens, resulting in an immune attack on the body’s tissues. The gastrointestinal microbiome has been involved in the emergence of autoimmune disorders, with dysbiosis contributing to pro-inflammatory and immune deregulation.^9^

Research has shown that variations in the components of the gut flora are related to autoimmune disorders and may influence the production of various. The interaction between gut microbes and the immune system predominantly occurs through Treg cells and Th17 cells, with Treg cells playing a vital anti-inflammatory role in autoimmune disorders.^85^ Furthermore, growing evidence suggests that gut microbial metabolites, such as lipopolysaccharides, peptidoglycan fragments, and teichoic acids, act as structural components of the microbiota,^33^ and short-chain fatty acids, tryptophan derivatives, bile acids, and polyamines function as substrates for metabolic processes and signaling molecules,^69^ significantly influencing host health and physiology. The role of gut metabolites extends beyond autoimmune disorders, as they have been found to impact a diverse range of diseases, including tumor, with both pro- and anti-carcinogenic effects.^26^

The intestinal flora has recently been recognized as a potential aspect of cancer resistance to the immune system.^54^ The growth and prevalence of various cancer types, particularly in sterile tissues and the epithelial barrier, are closely linked to microbiome activity. Carcinogenesis can occur locally or distantly in symbiotic ecosystems in the gut or other mucosa. The microbiome may promote cancer by producing carcinogenic substances or toxic metabolites or indirectly by inducing inflammation or immunosuppression.^39^ The complex interactions between specific intestinal metabolites and the development or suppression of tumor cell propagation are emerging as a new field in anticancer research.^26^ This review explores the gut microbiome’s impact on human health, emphasizing its role in metabolism, immune function, and disease. It also examines how gut microbial metabolites influence autoimmune disorders and the immunoediting process in solid tumors.

## Gut microbial metabolites and their role

Maintaining the gut barrier is critical for intestinal epithelial cells (IECs). Disruptions in its structure can trigger uncontrolled immune responses or allow unchecked microbiota growth, leading to various diseases. Tight junction proteins such as claudins, tricellulin, occludin, and zonula occludens (ZOs) are essential for preserving the barrier function by regulating cell-cell adhesion and controlling permeability in the intestinal epithelium.^3^ The interactions between IECs and immune cells, facilitated by microbial metabolites, are vital for sustaining homeostasis and gut barrier integrity, impacting overall host health and immune responses.^41^ However, biofilm formation in the gut can compromise the epithelial barrier’s integrity, leading to persistent inflammation, immune evasion, and diseases like cancer.^45^

Metabolites are small molecules that serve as byproducts or intermediates of metabolism.^65^ One key way the intestinal flora contributes to host physiological functions is by producing a wide range of metabolites and other small molecules.^71^ These metabolites, absorbable throughout the host’s digestive tract, can be detected in the bloodstream, often at levels comparable to or exceeding those of conventional drugs. Microbial molecules, even within a single metabolite, can have beneficial and harmful effects, varying their impact on several factors.^42^ For instance, LPS, a glycolipid found in the external membrane of Gram-negative bacterium, is a potent pro-inflammatory cytokine inducer and the best-characterized Toll-like receptors (TLR) ligand, with TLR4 playing a crucial role in detecting Gram-negative bacteria.^55^ Also, peptidoglycan in the intestines is essential to preserve gut architecture and microbiota balance and control gut homeostasis and composition.^17^ Immune tolerance in the gut is maintained by GALT, where innate immune cells distinguish pathogens from commensals via PRRs, activating CD4+ and CD8+ T cells. GALT-associated B cells produce IgA to prevent microbial penetration and limit harmful immune responses, aided by dendritic and T-follicular helper cells. However, molecular mimicry by microbial antigens can trigger T cell responses against autoantigens, especially in individuals with high-risk HLA genes, with gut barrier dysfunction and cross-reactive epitopes driving autoimmune activation.^22^ Gut microbes contribute to immune dysfunction through mechanisms like molecular mimicry, where microbial molecules share structural or sequence similarities with human components. This similarity disrupts immune tolerance, triggering prolonged inflammation and the proliferation of self-reactive immune cells that recognize cross-reactive epitopes on microbial and host antigens.^23^

**Figure 1.**
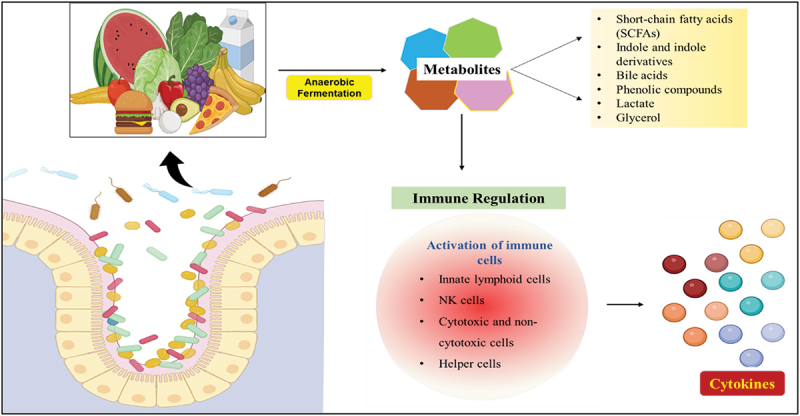
Gut microbial metabolites and their role.

The gut microbiota also produces numerous metabolites through the anaerobic fermentation of food in the colon. These metabolites maintain commensal diversity by inhibiting harmful bacteria as a defense mechanism. They play vital roles in immune regulation and host energy metabolism, and the human metabolome now includes thousands of microbial metabolites, many of which have yet to be fully understood. Different metabolic end-products the microbiome produces are utilized in distinct ways.^40^ The role of gut microbial metabolites is depicted in Figure 1.

The host immune system also depends on gut bacteria and their metabolites to trigger natural killer cells (NK), innate lymphoid cells, cytotoxic and non-cytotoxic lymphoid cells, and helper lymphoid cells. These interactions are critical for differentiating self from non-self in the early stages of life.^40^ A principal way the intestinal flora engages with the host is through its metabolites.^71^ The intestinal microbiota produces a wide variety of metabolites through the anaerobic fermentation of undigested dietary elements that reach the colon and endogenous compounds from both the host and the microorganisms. This metabolite repertoire significantly influences immune responses and disease risk. A monolayer of epithelial cells at the mucosal surface separates microorganisms from the host as a selective barrier, allowing microbial-derived metabolites to interact with host cells.^40^ Short-chain fatty acids(SCFAs), Indole-derived molecules, and secondary Bile acids(BAs) are among these essential metabolites. SCFAs, with six or fewer carbon atoms, are primarily produced from the breakdown of carbohydrates such as dietary fibers. Their role as central fermentation products accounts for their higher concentrations than other microbial metabolites.^56^

SCFAs are key regulators of intestinal immune cells and contribute to maintaining homeostatic immunity in systemic and mucosal compartments. Through regulatory T cells (Tregs) and a mechanism involving the Ffar2 receptor, SCFAs promote colonic immune balance. Additionally, SCFAs influence host metabolism and can directly prevent infections without affecting the host immune system.^80^ SCFAs work by inhibiting histone deacetylases (HDACs) and activating G protein-coupled receptors (GPCRs), two main signal transduction pathways. Regulating gene expression requires HDAC-mediated epigenetic changes. The development of several diseases, such as cancer, immune-related disorders, and metabolic disorders, is therefore impacted by SCFA-induced HDAC inhibition. Furthermore, SCFAs’ regulatory effects on inflammation, energy balance, and cellular signaling pathways are facilitated by the activation of GPCRs.^30^ By controlling cytokine production and restoring equilibrium between pro-inflammatory and immunosuppressive lymphocytes, SCFAs offer a novel therapy option for immune-mediated and autoimmune diseases. By affecting the function and differentiation of regulatory T cells (Tregs) and effector T cells, they can modify immunological responses, which highlights their potential for treating autoimmune diseases like multiple sclerosis, rheumatoid arthritis, and inflammatory bowel disease (IBD).^44^ In colitis models, butyrate has been demonstrated to reduce inflammatory responses by inhibiting HDAC activity, increasing Foxp3 expression, and Treg differentiation.^66^

Tryptophan (Trp), an essential aromatic amino acid obtained through diet, is metabolized through several pathways, with only a small portion used for protein synthesis. The rest is processed by endogenous host cells (through the kynurenine and serotonin pathways) or gut microorganisms, which produce indole and its derivatives.^16^ These indole derivatives have been widely explored for their antifungal, anti-platelet, and antioxidant effects in multiple diseases. They are fundamental in maintaining the intestinal barrier function and immunity. By acting as ligands for the aryl hydrocarbon receptor (AhR), a transcription factor in immune response cells, indole and its derivatives regulate intestinal immune homeostasis by influencing the associations between gut microbiota and the host’s innate defense system.^2^ The gut microbiota’s tryptophan metabolism produces indole derivatives, essential ligands for the aryl hydrocarbon receptor (AhR), and supports intestinal immunological homeostasis and barrier function.^24^ Dysbiosis and decreased expression of AhR hinder AhR activation, disturb the barrier, change immunological responses, and encourage chronic inflammation in autoimmune illnesses such as IBD. The therapeutic promise of AhR and its microbial ligands in IBD is highlighted by murine research demonstrating that AhR deficiency exacerbates colitis and that IBD patients, particularly those with Crohn’s disease, have decreased AhR expression in immune cells.^43^

Bile acids (BAs), derived from cholesterol, are central in assimilating nutrients, glucose regulation, and energy balance. The liver synthesizes initial BA, such as chenodeoxycholic and cholic acids, which get secreted into the duodenum as taurine or glycine conjugates to emulsify dietary fats and vitamins that dissolve in fats(fat-soluble). Microbial enzymes deconjugate these BA within the small intestine using bile salt hydrolases. Most BAs (over 95%) are resorbed in the distal ileum and recycled through enterohepatic circulation, while less than 10% escapes reabsorption, reaching the colon where bacteria convert them into secondary and free BA.^21^ Beyond their digestive role, BAs and their metabolites influence immune regulation. They interact with immune cells such as dendritic cells, macrophages, regulatory T cells (Tregs), Th17 cells, myeloid-derived suppressor cells (MDSCs), innate lymphoid cells (ILCs), CD4 and CD8 T cells, B cells, and natural killer T (NKT) cells to affect the differentiation and function of these immune cells, thereby ensuring the maintenance of both gut and systemic immune balance.^81^ Disruption of BA homeostasis is associated with immune-related disorders, including gut diseases. Since the gut microbiota metabolizes BAs, modifications to their makeup affect the pathways that BAs mediate, including Farnesoid X Receptor (FXR) signaling.^67^ Bile acids cause host cell receptors such as TGR5 and FXR to become active. FXR regulates the expression of antimicrobial peptides, which affects gut microbiota, whereas TGR5 affects intestinal motility and energy expenditure. By altering bile acids and influencing their recycling and reabsorption in the circulation of the enterohepatic region, gut bacteria highlight the significance of bile acid dynamics and microbial metabolism.^74^

## Mechanisms linking gut microbiome to autoimmunity

### Role of dysbiosis

The gut microbiota, which forms a complex community of microorganisms, can establish symbiotic or potentially pathogenic relationships with the host. These microbes play a direct and indirect role in maintaining homeostasis within the human body, significantly impacting overall health. However, disruptions in this microbial balance can result in dysbiosis, a general alteration in the composition of physiological microbial communities, which has been associated with various diseases. This loss of the mutually beneficial relationship between human cells and the microorganisms that coexist with us is a hallmark of dysbiosis.^15^

Infections, diet, exercise, sleep patterns, antibiotic exposure, and co-morbidities can contribute to gut microbiota instability.^53^ Dysbiosis, broadly referring to the imbalance of gut microbes linked to adverse health effects, can lead to the breakdown of immune tolerance, excessive T cell activation, and an increase in pro-inflammatory cytokine production. These processes can trigger autoimmune responses and affect the onset of autoimmune disorders.^10^ The effects of dysbiosis extend beyond gastrointestinal disorders, such as obesity, colorectal cancer, inflammatory bowel disease, and enteric infections. It has also been linked to immunological conditions outside the gut, including allergies, eczema, asthma, and autoimmune disorders affecting distant organs, such as type 1 diabetes(T1D), immune-mediated arthritis, and experimental autoimmune encephalomyelitis.^37^

**Figure 2.**
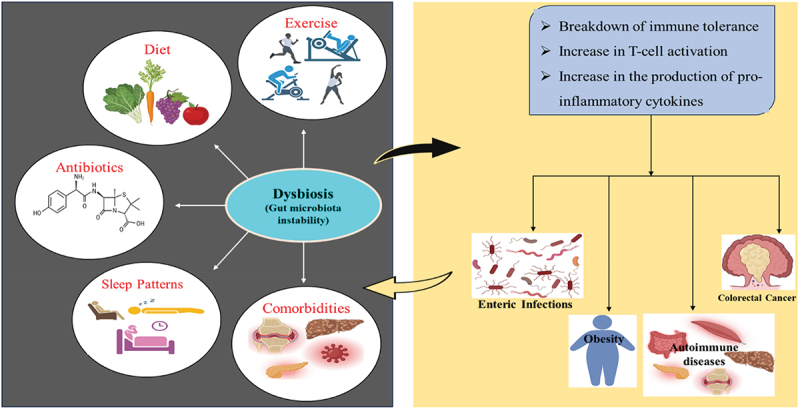
Relationship between gut dysbiosis and host factors.

The mucosal or epithelial barrier may become permeable when the gut microbial community becomes disrupted. This increased permeability allows microbiota-derived byproducts to enter the systemic circulation, potentially influencing distant organs, including the central nervous system.^19^ Figure 2 illustrates the interplay of dysbiosis and its associated factors.

### Impact of gut metabolites on autoimmune disorders

The study of gut flora and its impact on human health has gained significant attention in recent years. Shifts in the gut microbiota composition have been connected to the emergence of various autoimmune disorders, including T1D, Rheumatoid arthritis(RA), Systemic Lupus Erythematosus(SLE), and Inflammatory Bowel Disease(IBD), such as Crohn’s Disease (CD) and Ulcerative Colitis (UC). Additionally, autoimmune skin disorders like atopic dermatitis, vitiligo, psoriasis vulgaris, and autoimmune disorders neurological disorders have also been connected to alterations in gut microbiota composition.^47^ For example, fluctuations in SCFA levels, such as acetate, butyrate, and propionate, can significantly impact islet β-cell autoimmunity in T1D. These SCFA types also stimulate the production of Tregs and B-cell antibodies.^58^ IgA and IgA+ B lymphocytes are vital in maintaining intestinal homeostasis and influencing proneness to autoimmune disorders. Nonetheless, the exact mechanisms through which the IgA immune response to modified gut microbiota and SCFA creation impacts individuals with T1D are still unclear.^64^

In RA, microbiota-modified BA is key in disease progression. Increased BA levels in RA patients have been found to decrease erythrocyte sedimentation rate (ESR), RF-IgA anti-CCP antibodies, RF-IgM, and RF-IgG and reduce the number of swollen and painful joints. In the advancement of rheumatoid arthritis (RA), pro-inflammatory cytokines like GM-CSF, IL-18, IL-21, IL-1β, IL-2, IL-7, IL-33, IL-17, and IL-23 contribute to the disruption of the intestinal epithelial barrier and triggering inflammatory reactions. Furthermore, secondary BA, such as lithocholic acid (LCA) and deoxycholic acid (DCA), Are proficient in reducing TNF-α production by macrophages by activating the farnesoid X receptor (FXR).^75^

### Molecular pathways

Innate immune cells such as dendritic cells, monocytes/macrophages, and natural killer cells have pattern recognition receptors (PRRs), which mediate communication between the host and pathogens. TLRs, RIG-I – like receptors (RLRs), C-type lectin receptors (CLRs), and NOD-like receptors (NLRs) are among the families of PRRs that identify microbe- or pathogen-associated molecular patterns (MAMPs or PAMPs).^82^ The mucosal immune system employs several mechanisms to recognize the microbiota and its metabolites, including purinergic receptors, TLRs, aryl hydrocarbon receptors (AHR), G protein-coupled receptors (GPCRs), and NLRs. When these receptors are activated, they often lead to cytokine or chemokine production, which is carefully regulated by factors such as the type of cell expressing the receptor, the strength of the signal, tissue location, and the surrounding microenvironment. These sensors initiate innate immune responses to control the microbiota: TLRs stimulate the release of antimicrobial peptides (AMPs), GPCRs promote T cell activation, AHR induces IL-22 production, MyD88 regulates microbiota-specific IgA secretion, and NLRP6 triggers mucus secretion.^28^ The main components of microbial macromolecules that cell surface TLRs can identify are proteins, lipids, and lipoproteins. For example, Lipopolysaccharide (LPS) is recognized by TLR4. In contrast, glycosylphosphatidylinositol-anchored mucin-like glycoproteins from *Trypanosoma cruzi* trypomastigotes, lipoteichoic acids, lipoproteins, zymosan, peptidoglycans, and mannan are among the pathogen-associated molecular patterns (PAMPs) that TLR2 combines with either TLR1 or TLR forms a heterodimer. Conversely, TLR5 can identify bacterial flagellin. By activating TLRs, which increase APC signaling and encourage T cell activation, microbe-derived adjuvants improve the synthesis of cytokines, the expression of costimulatory molecules, and the antigen-presenting function in APCs. TLRs are, therefore, essential for connecting cell-mediated and innate immunity.^1^ In autoimmune diseases, the immune response is thought to result from molecular mimicry between pathogen-derived antigens and self-antigens or nonspecific activation of the innate immune system, leading to a decline in immunological tolerance. This triggers the production of T cells and antibody responses against self-antigens, with Toll-like receptors (TLRs) implicated in numerous autoimmune diseases.^31^ In cancer, the TLR2 agonist acGM-1.8, mimicking pathogen-associated molecular patterns, has been shown to effectively reprogram macrophages to exhibit anti-tumor activity while reactivating T cell-mediated immunity, underscoring the therapeutic potential of targeting TLR pathways and leveraging antigen mimicry for cancer treatment.^35^

Tregs are decisive for downregulating immune responses and inflammation, acting as key regulators of peripheral tolerance. Tregs are produced in the thymus (natural Tregs or nTregs) and peripheral tissues (induced Tregs or iTregs). Their development is influenced by two key anti-inflammatory cytokines, TGF-β and IL-10. An environment rich in TGF-β encourages the accumulation of Tregs, while IL-10 facilitates a positive feedback loop that enhances further Treg differentiation.^4^ In contrast, Th17 cells are essential for defending mucosal surfaces from pathogens such as bacteria, fungi, and viruses. These cells are especially abundant in the lamina propria associated with the small intestine and are distinguished by their production of IL-17, which triggers tissue inflammation. Despite their contrasting roles, Tregs and Th17 cells share common regulatory pathways, including transcription factors and cytokines, which regulate their development, akin to other T-helper cell subsets.^68^

## Microbial metabolites and cancer immunoediting

The immune system has a twofold function, both hindering and, paradoxically, facilitating the development and advancement of tumors. Cancer immunoediting starts after cellular transformation, with the potential to eliminate transformed cells. Nevertheless, if the immune system fails to eradicate the tumor, the cancer may enter a phase of immune-mediated equilibrium. In this phase, the immune system regulates the tumor, influencing its immunological traits, which may result in a suppressive tumor microenvironment (TME) that allows tumor escape and further growth. This continuous immunoediting occurs during tumor development and progression and in patients undergoing cancer immunotherapy, where the treatment impacts the ongoing immunoediting process.^51^

### Immunoediting phases and microbial influence

**Figure 3.**
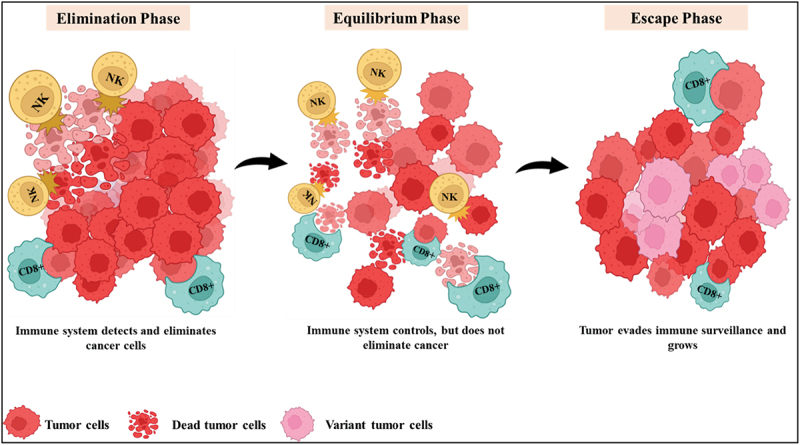
Stages of cancer immunoediting.

The cancer immunoediting hypothesis suggests that the immune system is essential for managing tumor growth and affecting the tumor’s characteristics. This concept outlines three distinct phases in the interaction between the immune system and the tumor: elimination, equilibrium, and escape.^50^ In the elimination phase, the innate and adaptive immune systems collaborate to identify and eradicate malignant or transformed cells before they become detectable. The equilibrium phase is marked by a state of balance where the immune system cannot eliminate the tumor cells, yet those tumor cells do not entirely escape immune surveillance. In the escape phase, tumor growth and proliferation occur unchecked by the immune system, depicted in Figure 3. The accumulation of rapidly dividing tumor cells, alongside other stromal components, contributes to creating a more complex and immunosuppressive tumor microenvironment, further disrupting the balance between tumor cells and immune defense mechanisms. Throughout the cancer immunoediting process, the immune system’s capacity to detect, recognize, and eliminate tumor cells is crucial for preventing tumor progression.^18^

Examining extensively annotated cancer datasets has uncovered unique microbial signatures in tissues and blood across various cancer types. However, the exact role of microbiota in cancer progression remains ambiguous, as it can either promote or inhibit tumor development.^29^ Garret et al. (2015) emphasized the intricacy of this relationship by identifying three main mechanisms through which microbiota may contribute to carcinogenesis: (i) regulating the balance between cell proliferation and apoptosis, (ii) influencing the immune response, and (iii) affecting the host’s metabolism. A notable instance of bacteria-induced cancer is the CagA protein toxin produced by *Helicobacter pylori*. Furthermore, *Helicobacter hepaticus* has been found to facilitate hepatocellular carcinoma and mammary carcinomas and speed up the development of intestinal adenomas in mouse models. *H. hepaticus* releases a cytolethal distending toxin that induces DNA double-strand breaks, which, combined with chronic inflammation, leads to carcinogenesis in the lower bowel, as demonstrated in murine studies.^52^ A similar mechanism of tumor promotion is observed with the colibactin toxin produced by *Escherichia coli*. Likewise, *Shigella flexneri* generates the cysteine protease-like virulence gene A (VirA), which disrupts the DNA damage response, and inositol phosphate phosphatase D (IpgD), which degrades the host p53 and alters cell cycle regulation.^46^ Gut bacteria directly interact with host cells and impact host biology through microbial-derived metabolites. Understanding the roles of SCFAs and polyamines and their effects on colon carcinogenesis and the TME underscores the significance of microbiota-metabolite-cell interactions.^36^

Microorganisms exhibit a remarkable capacity to metabolize substantial amounts of chemotherapeutic agents. For instance, gut microbiota-derived metabolites such as propionate, kynurenic acid (KYNA), and indole-3-carboxaldehyde protect patients from the adverse effects of radiotherapy, improving survival rates. Additionally, a combination of microbial metabolites plays a significant role in cancer treatment,^6^ with butyric acid produced by gut microbial metabolism improving T-cell receptor signaling and promoting cytokine generation with anticancer effects. This enhances immune cell anticancer activity.^49^ Furthermore, many studies have mentioned that the therapeutic strategies targeting enzymes involved in acetate metabolism, such as ACSS2 or modifying acetate levels, may strengthen T cell responses and improve cancer immunotherapy outcomes.^70^

## Interplay between microenvironment of autoimmunity and tumor immunoediting

Various cell types in the microenvironment of inflamed tissues release a diverse range of mediators, such as lipid mediators, chemokines, cytokines, growth factors, ROS, RNS, neuropeptides, and remodeling enzymes. These mediators regulate the recruitment of new cells to the site of inflammation and their interactions. Both tissue and stromal cells produce these mediators. While their effects are localized within the tissue, they can also impact distant organs, such as influencing premetastatic sites in cancer or extending autoimmune processes to multiple organs. Shared features of autoimmune and cancerous microenvironments are discussed below, focusing on the role of gut microbiota as a convergence point.

### The microenvironment of hypoxia

Hypoxia is characterized by low oxygen levels and is observed in all inflammatory tissues. HIFs are crucial regulatory transcription factors orchestrating the cellular adaptive response to hypoxia by enhancing glycolysis, promoting angiogenesis, increasing leukocyte infiltration, and inducing immunological suppression,^27^ pivotal in cancer and autoimmune diseases. In cancer, uncontrolled tumor cell proliferation depletes oxygen and nutrient supplies, stimulating angiogenesis primarily through hypoxia. Various oxygen tensions are observed within tumors, with almost 50% of advanced solid tumors showing partial pressure oxygen values below 5 mmHg.^7,90^ Hypoxia further promotes glycolysis in tumor cells, even under normoxic circumstances, known as the Warburg effect.^48^ In autoimmune disorders, increased leukocyte infiltration raises oxygen demand beyond availability, resulting in hypoxia.^8^ For example, systemic lupus erythematosus (SLE) anemia contributes to tissue hypoxia, with elevated proangiogenic factors like TNF, VEGF, and TGF reported in patients.^32,87^ Also, recent evidence indicates that gut microbiota influences the hypoxic response by modulating HIF stability through microbial metabolites such as butyrate. This interaction maintains gut barrier integrity and links hypoxia with angiogenesis in cancer and autoimmune diseases, highlighting the microbiota’s central role in shaping the microenvironment.^84^

### Macrophage and neutrophil interactions in cancer and autoimmune disorders

Macrophages and neutrophils are versatile immune cells that play significant roles in cancer and autoimmune disorders. In cancer, tumor-associated macrophages (TAMs) frequently polarize to an M2-like state, releasing anti-inflammatory cytokines like IL-10 and angiogenic factors that promote tumor growth. Neutrophils, classified as tumor-associated neutrophils (TANs), also exhibit pro-tumoral phenotypes, releasing factors like MMP-9 that facilitate angiogenesis.^57,86^ In autoimmune disorders, macrophages often display mixed phenotypes, while neutrophils release ROS, proteases, and neutrophil extracellular traps (NETs), exacerbating inflammation.^83^ These interactions amplify inflammation and tissue damage, shaping the microenvironment in both conditions. Gut microbiota impacts these interactions by modulating macrophage and neutrophil activity through microbial metabolites, further influencing the immune response in cancer and autoimmunity.^72^

### Bridging autoimmunity and cancer development by autoantibodies

Autoantibodies are critical in both autoimmune disorders and cancer, marking cells for destruction and influencing cell signaling. In autoimmune diseases, autoantibodies contribute to pathogenesis and serve as diagnostic markers, such as anti-dsDNA antibodies in SLE and anti-insulin antibodies in T1D.^20^ In cancer, autoantibodies target tumor-associated antigens (TAAs), initially contributing to tumor cell death but later adapting to the immunosuppressive tumor microenvironment. This dual role underscores their significance in both conditions.^88^ Gut microbiota may influence autoantibody production by modulating immune tolerance and neoantigen presentation, further linking autoimmunity and cancer.^11^

### Role of intratumoral microbiota

The gut microbiota has been suggested as a source of intratumoral microbiota, which plays a crucial role in tumor progression. Intratumoral microorganisms remodel the tumor immune microenvironment by interacting with immune cells and promoting angiogenesis through bacterial components like lipopolysaccharides (LPS).^20^ This interaction triggers cytokine and chemokine release, supporting angiogenesis and immune evasion in the tumor microenvironment. Intratumoral microbes also influence tumor metabolism, immune responses, and overall progression, creating a link between the microbiota and cancer development.^38^ Additionally, intratumoral microbes may interact with autoantibodies, further modifying the tumor microenvironment and promoting tumor growth.^73,77^ These interactions underscore the intricate links between microbiota, autoimmunity, and cancer. Shared mechanisms such as hypoxia, angiogenesis, immune cell interactions, and autoantibodies underscore their convergence within the microenvironment. Emerging evidence reveals the pivotal role of gut and intratumoral microbiota in shaping these processes, offering potential therapeutic strategies targeting microbiota to manage both conditions. The Gut Microbiota’s Role in Connecting Autoimmunity and Cancer Mechanisms is represented in Table 1.

**Table 1.** Gut microbiota’s role in connecting autoimmunity and cancer mechanisms.

Mechanism	Autoimmunity	Autoimmune Relevance	Role of Gut Microbiota
Hypoxia	Increased leukocyte infiltration raises oxygen demand, leading to tissue hypoxia (e.g., anemia in SLE).	Tumor cell proliferation creates hypoxic microenvironments, triggering angiogenesis and the Warburg effect.	Microbial metabolites like butyrate stabilize HIF, linking gut barrier integrity, hypoxia, and angiogenesis.
Macrophage and Neutrophil Activity	Mixed macrophage phenotypes and neutrophil extracellular traps (NETs) amplify inflammation in autoimmune disorders.	Tumor-associated macrophages (M2-like) and tumor-associated neutrophils promote angiogenesis and immune evasion.	Microbial metabolites regulate macrophage and neutrophil activity, modulating immune responses in both conditions.
Autoantibody Production	Autoantibodies target self-antigens, contributing to pathogenesis (e.g., anti-dsDNA in SLE).	Autoantibodies initially target tumor-associated antigens but later adapt to promote immune evasion.	Gut microbiota influences autoantibody production via neoantigen presentation and modulation of immune tolerance.
Intratumoral Microbiota	Not directly observed in autoimmunity but microbiota can indirectly influence tissue inflammation and immune responses.	Intratumoral microbiota remodels the immune microenvironment, triggering angiogenesis and immune evasion through microbial components like LPS	Gut microbiota serves as a source of intratumoral microbiota, influencing tumor metabolism and immune responses.

## Conclusion

The gut microbiome and its metabolites are essential in regulating immune responses and affecting autoimmune disorders and cancer onset. The complex interactions between microbial metabolites like SCFs, secondary BA, and indole derivatives and immune function underscore the significance of sustaining a balanced gut microbiota for overall health. Dysbiosis disrupts immune tolerance and contributes to systemic inflammation and disease progression. Additionally, the common microenvironmental characteristics of autoimmune and malignant tissues highlight the importance of understanding the underlying inflammatory processes. The involvement of autoantibodies in both conditions offers a chance for cross-disciplinary insights that could improve our understanding of immune dynamics. Ultimately, further research is essential to elucidate the specific mechanisms underlying these interactions and to identify potential therapeutic approaches targeting the gut microbiome. By advancing our understanding of these complex relationships, we can develop innovative strategies to prevent and treat autoimmune disorders and cancer, paving the way for improved patient outcomes.

## Data Availability

Data sharing is not applicable to this article as no new data were created or analyzed in this study.
